# Evolution of MSCP-Enabled Healthcare Ecosystem: A Case of China

**DOI:** 10.1155/2021/1167221

**Published:** 2021-08-23

**Authors:** Yang Liu, Liwei Tian

**Affiliations:** School of Information Engineering, Shenyang University, Shenyang 110044, China

## Abstract

This paper considers a case that Chinese healthcare organizations in China leverage mobile smart cloud platform (MSCP) to build a self-sustaining healthcare ecosystem, which presents an evolutionary process of three phases. Multiple elements have been involved in the system, which has reinforced one another. Finally, an ecosystem is formed, which is economically sustainable and scalable nationwide. The study adds to the understanding of the ecosystem-based approach to address complex problems. It offers important implications for designing healthcare MSCPs which may help transform the healthcare industry.

## 1. Introduction

As is well known, China is the most populous country in the world as well as one of the fastest-growing markets in the world. At present, China's healthcare industry is facing many problems that other developed countries faced in the past or are even facing to this day. In terms of China's social environment, the issue of an aging population is worsening (China is the only country with more than 100 million elderly persons and the largest aging population in the world), the number of people with chronic diseases continues to increase, and people's health management awareness has been increasing as well. In the industrial environment, the high concentration of high-quality medical resources is concentrated in few big cities. The whole country faces the challenges of unbalanced structure of healthcare service system, inefficient implementation of graded medical services, shortage of healthcare workers, increasing medical costs, and inefficient risks of implementing national medical insurance system which have exacerbated the existing crisis in China's healthcare service industry. There are information and communication technologies (ICTs) and Internet-enabled solutions to these issues. As an example, the mobile smart cloud platform (MSCP) has been used to improve healthcare services, including, e.g., in professional online healthcare treatment, health counseling, and telemedicine services for outpatients.

In the 1980s, countries like the UK started to promote the use of Internet-based technologies in healthcare. As an example, a doctor can track and provide consultation to his patients equipped with intelligent wearable devices when locating at home or in public places [1]. In October 2014, China's first online hospital was born in Guangdong Province. Since then, the Internet healthcare entrepreneurs have mushroomed. In just one year until the end of 2016, more than 10 online hospitals have emerged, some with different labels like cloud hospitals and Internet hospitals. The fast growth of Internet healthcare industry in China is based on a supportive policy and technology environment; Internet healthcare services have been included in the “13th Five-Year Plan” as an industrial organization; “smart healthcare” is the use of digital, visual mode, vital sign collection, and health monitoring to achieve the sharing and rational distribution of healthcare resources. The information and communication technologies (ICTs) and Internet technologies including sensor technology, wearable smart devices like mobile phones, mobile and WiFi technologies, deep learning technologies, big data, and cloud computing enable quick access to a broader range of information and services.

This paper focuses on MSCP in healthcare from the ecosystem perspective. The ecosystem concept was initially used to analyze the applications of ICTs, which involved the collaboration of a broad range of actors [2]. In recent years, the term of service ecosystem is introduced, academics insist on service-led (SD) logic, placing services at the core of ecosystems, which is composed of social actors and their respective resources, linked together by value propositions across a network of relationships [3, 4]. Within this system, stakeholders form a unitary, interrelated, collaborative, and competitive community which promotes the added value of products and services. Healthcare represents a service ecosystem which included multiple social actors and is more complex than a simplistic consideration of the doctor-patient model. The healthcare service ecosystem is composed of heterogeneous and complementary entities such as patients, doctors, equipment suppliers, pharmaceutical companies, insurance companies, and public health authorities [5, 6]. They form an interconnected and interdependent organism. Each actor in the ecosystem exerts its core strengths and works together with others to sustain the system. The system is dynamic. It evolves by phases and finally achieves sustainability. A broader perspective is also necessary to understand how ecosystems evolve, which takes into account all the roles and interrelationships involved in the infrastructure, policies, and regulations required, promotion mechanisms and sustainability approaches, cultural values, interconnections with external factors, and so on.

This article focuses on MSCP-enabled ecosystem in healthcare, a kind of service ecosystem, which takes the healthcare cloud service as the core asset, connects the stakeholder of healthcare through internet technology, forms a virtual organism that interacts with each other and promotes each other, the social actors that depended on each other, seeks the dynamic balance of the system in the competition, and finally realized the value increment of the whole system. Through a case study, we will address such a research question: at the macro-, meso-, or microlevels, how do relevant societal actors collaborate to use MSCP to form a healthcare ecosystem?

## 2. Research Methods

To understand the evolution of healthcare service ecosystems, we consider a pioneering case of MSCP based healthcare project in China. A case study design is proper for addressing how to type research question.

The research originated from the author's participation in the healthcare project, which was initiated by the healthcare authority of A City, and entrusted B Company and P University to develop a mobile smart cloud platform (MSCP) in the way of school-enterprise cooperation. Company B is willing to share the relevant information of the smart healthcare project. City A is a second-tier city located in the south-central region. The rural population is mostly, and the city's information base is weaker than that of developed cities along the eastern coast. In order to test the MSCP platform, the government healthcare administrations in City A established 24 small-scale healthcare clinics and identified 6 medium-scale basic hospitals and 1 large-scale and high-level hospital as pilot healthcare institutions. Through the analysis of the case, we can explore the use of a mobile intelligent cloud platform to build a self-sustaining healthcare ecosystem for similar cities in China. In order to solve the problems of reducing healthcare costs, making up for healthcare shortcomings, adjusting the structural distribution of healthcare resources, etc., the first experience and revelation are provided. To collect data, we carried semistructured interviews with sponsors and leaders of the project to develop a preliminary understanding of the various stages through which MSCP has progressed. The interviews help us to understand the key actors in the healthcare ecosystem, including patients, healthcare service providers, health insurance agencies, and government healthcare administrations. Among them, the healthcare service providers include the local doctor in the small-scale healthcare clinics, online doctors in the medium-scale basic hospitals, the specialist doctors in the large-scale and high-level hospitals, pharmaceutical companies, healthcare equipment companies, and third-party developers. Before each interview, the researcher would carefully design an outline according to relevant research questions and who the interviewee was. After the outline was laid out, the interview was recorded for future analysis. After the interview was conducted, the recorded file would be transcribed into a text summary and issued an identification number. The field interview process began in January 2017 and concluded in December 2017, covering 28 interviewees. The interview arrangements are illustrated in Table 1. In the process of data analysis, we kept contact with interviewees and would consult them when any concerns emerged.

Except for interviews, there are mainly two data sources: internal documents including the project implementation plan and project process documentation; data from online channels such as the government authorities' websites and news media. In order to improve the reliability and validity of the research results [7], researchers implemented a triangulation approach to cross-validate key information from multiple sources.

## 3. The Emergence of an MSCP-Enabled Healthcare Ecosystem

**Figure 1:**
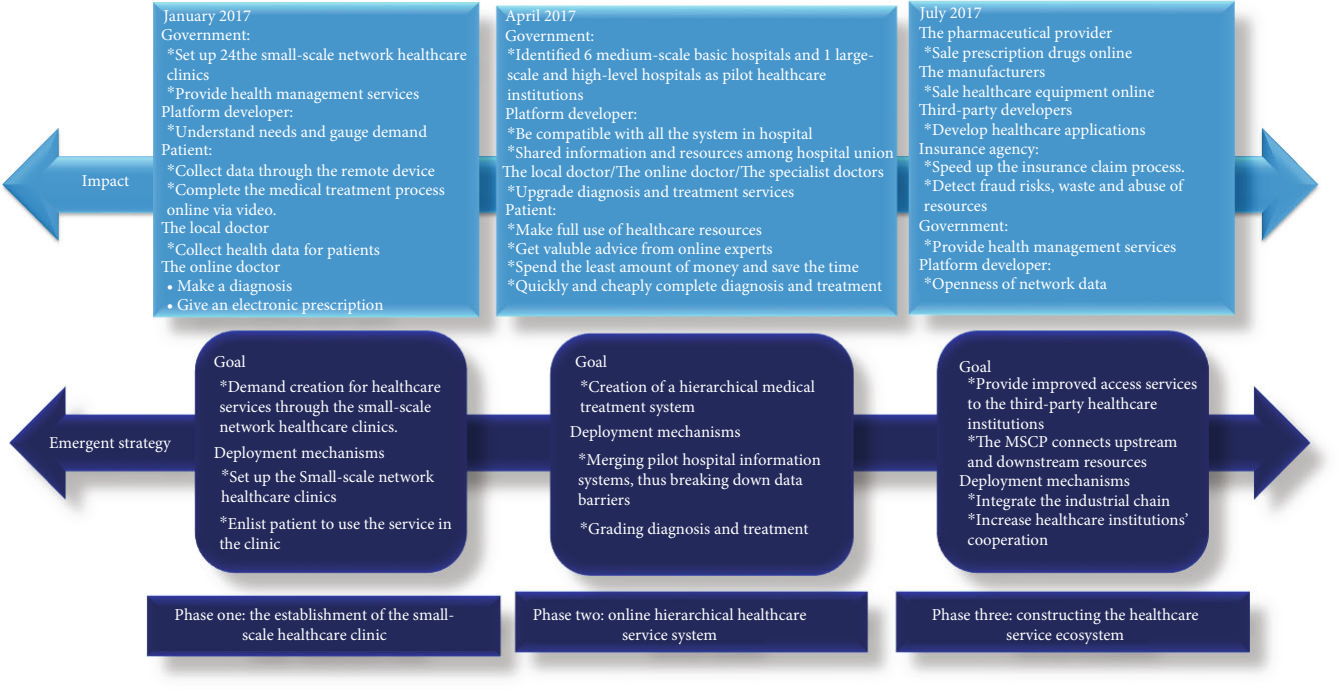
Evolution of MSCP-enabled ecosystem.

The case study results are summarized in Figure 1. The healthcare service ecosystem supported by MSCP has progressed through three significant phases since its inception. In the first stage, it assessed the existing demand for technology-enabled services to establish MSCP, and the small-scale network healthcare clinics are being built to complete the information collection and transformation for MSCP. The second phase expanded the service coverage to a larger area through integrating the small-scale network healthcare clinics, the medium-scale basic hospitals, and the large-scale and high-level hospitals, which established a hierarchical healthcare treatment pattern by MSCP. In the third phase, participants from diverse and complementary sectors in the healthcare field were also integrated into the platform ecosystem to develop collaboratively.

### 3.1. The First Stage: The Establishment of the Small-Scale Healthcare Clinics

In January 2017, MSCP engaged with the doctor in the medium-scale basic hospitals and all the patients to understand their needs and gauge demand for the platform. In this stage, the doctors in 6 medium-scale basic hospitals can diagnose online by the MSCP platform, and 24 small-scale healthcare clinics were established in urban residential communities and rural areas according to the geographical division. The telemedicine equipment was set up, with the help of the local doctor in the small-scale healthcare clinics, patients used telemedicine equipment to collect health data and complete the process of seeking healthcare treatment directly with online doctors in the medium-scale basic hospitals by video. This new healthcare pattern diagnosed diseases through remote consultation, remote ECG, remote imaging, and remote inspection and gives an electronic prescription online; at the same time, a personal electronic health archive was established. Services provided by small-scale healthcare clinics to patients through the platform include generation and cancellation of appointments online, telemedicine, home health monitoring, self-care, health training, health management, first aid nursing, and public health information. Patients can receive health check-ups via the small-scale healthcare clinics as well. In the same time, the MSCP's extended functions include treatment through prevention and rehabilitation of chronic diseases including providing health assessments, health interventions, and health promotion services. Customer-downloaded apps may be used to guide patients with reasonable exercise, diets, accommodations, and so on.

Technically, the small-scale healthcare clinics are characterized by the large number and wide geographic distribution. Due to the small flow of patients and the small amount of data in a single small-scale healthcare clinic, the SSL VPN is the simplest and safest solution to the remote user access data. The medium-scale basic hospitals are characterized by the small number and a larger flow of patients. Due to a large amount of data, the connection stability requirements, the IPSec VPN is the main mode of connection, which is a relatively complete system of VPN technology and provides a series of protocol standards.

In the first stage, MSCP provides technology to extend the healthcare service of the medium-scale basic hospitals to the small-scale healthcare clinics through MSCP. The government healthcare administrations in City A established 24 small-scale healthcare clinics. Patients can be treated directly by the doctor online via video. The local doctors in the small-scale network healthcare clinics help patients collect and monitor all kinds of health indicators through physical examinations and offer daily health management services, general health management, and disease prevention for a subhealth population. The online doctors in the medium-scale basic hospitals undertake outpatient clinical services online, make a diagnosis, and give an electronic prescription according to the patient's own description of the illness, the test results collected by telemedicine equipment. Patients and doctors establish cooperative relationships to cope with the disease and complete the online diagnosis and treatment process.

As a result, the treatment of common diseases, frequently occurring diseases, and chronic diseases can be achieved online at local small-scale healthcare clinics. 80-90% of patients with diseases can be treated remotely online via the platform, without going to the large-scale and high-level hospitals.

In village clinics, I can communicate with a doctor from the network hospital conveniently. I do not need to worry about to travel to a faraway big hospital for a small health problem. (Patient)

The patients can be diagnosed and treated in their own community hospital and directly take needed medicine or injections, they can skip going to a big hospital to wait in line and conduct various tests, which saves the time and energy. (Online doctor)

The patients suffering from chronic diseases such as diabetes mellitus and hypertension can conduct real-time examinations directly from community hospitals and collect health indicators to complete the referral rehabilitation without traveling to a large hospital far away. (Online doctor)

### 3.2. The Second Stage: The Establishment of Online Hierarchical Healthcare Service System

In April 2017, the large-scale and high-level hospitals were connected to the MSCP in this stage. The online hierarchical healthcare service system was established based on MSCP, which consisted of with the small-scale network healthcare clinics, the medium-scale basic hospitals, and the large-scale and high-level hospitals, which established the division and cooperative relations, and healthcare resources could be reasonably distributed. Patients can be registered into the system, and prediagnosis will be based on the patient's conditions when finding a suitable healthcare service provider. After the patient's initial visit, they will receive diagnostic results back online and complete their referral remotely. The hospital decentralization has been promoted, and the patient resources of big hospitals have been diverted according to the graded diagnosis and treatment system. Only patients with critical illness need to be hospitalized at the large-scale and high-level hospitals, thereby relieving workloads in the large-scale and high-level hospitals, which can concentrate on research and healthcare services for difficult diseases. The MSCP can provide data sharing and assist hospitals at all levels to implement linkages throughout the network, including remote consultation and healthcare service/supply exchanges. In-person healthcare services would be extended to all levels of health institutions' network via the Internet and a proprietary app.

Technically, the healthcare information system of every given pilot hospital will be transformed to accommodate the MSCP. One common information standard should be constructed to merge pilot hospital information systems and break down data barriers. Existing healthcare information systems were independently constructed by major hospitals. They need to conform to a new standard so that data would be transferable throughout the new system. Process management through business process reengineering and optimization is conducted. Issues arising from discrepancies in personnel, assets, business management, patient service, and online interaction with healthcare insurance agencies between each location have been addressed. Utilizing data exchange technology, the original HIS, LIS, PACAS, and EHR systems of the pilot hospitals were connected to the MSCP. Based on unified data standards, the data could be merged successfully. Information and resources may be effectively shared among all-level hospitals. The large-scale and high-level hospitals are also characterized by a relatively small number and a larger flow of patients. Due to a large amount of data, taking into account the demand for image data sharing, the flow is 5 to 10 times of the general small-scale healthcare clinics and the medium-scale basic hospitals; the connection mode is dominated by MPLS VPN. MPLS VPN refers to the use of MPLS technology to build enterprise IP network to achieve cross-regional, security, high-speed, reliable multiservice high-quality communication methods.

In the second stage, the government healthcare administrations in City A identified 6 medium-scale basic hospitals and 1 large-scale and high-level hospital as pilot healthcare institutions. MSCP provides technology to be compatible with all the systems in all-level hospitals and establishes the online hierarchical healthcare service system. For rare cases of illness, the specialist doctors in the large-scale and high-level hospitals would be responsible for the diagnosis and treatment of critically ill patients and the guidance of basic hospitals and the patients can get valuable advice from well-known experts in the large-scale and high-level hospitals. The patients could spend the least amount of money and time to make full use of healthcare resources of the online hierarchical healthcare service system. The doctors in all levels of hospitals aim to achieve up and down linkages and upgrade diagnosis and treatment services for the patients.

The patients can make full use of the large-scale and high-level hospitals and expert medical staff throughout the network to share healthcare resources. (Local doctor)

For rare cases of illness, the patients can get valuable advice from well-known experts in the large-scale and high-level hospitals. (Online doctor)

I can quickly and cheaply choose the best diagnosis and treatment using the online system. (Patient)

Technically, the healthcare information system of every given pilot hospital will be transformed to a given hospital's healthcare information system which is usually built internally by the hospital, causing such systems to be incompatible with one another. A comprehensive system would improve the overall work efficiency of healthcare staff and managers. (Developer of the SMCP)

The establishment of unified data standards is conducive to the sharing of information and resources of the online hierarchical healthcare service system. (Developer of the SMCP)

### 3.3. The Third Stage: Constructing the Healthcare Service Ecosystem

In July 2017, the MSCP provided improved access services, software services, hardware services, and platform services to the third-party healthcare institutions, integrates the hospital resources, and gradually opens up the upstream and downstream resources such as pharmaceutical provider, manufacturers of the healthcare equipment, third-party developers of healthcare cloud services, healthcare insurance agencies, and government health administrations. The functions of MSCP transformed traditional professional solutions to the healthcare service ecosystem step by step. The industrial chain is integrated into the system to improve the wealth of wisdom within the healthcare service platform's service portfolio and increase healthcare institutions' cooperation in the field of development.

The drug and the equipment decentralization have been pushed forward; the process of medicine, healthcare equipment, and diagnostics are separated. MSCP also provides medical big data for government healthcare administrations and healthcare insurance agencies. The medical big data obtained through personal electronic health archive include drug data, clinical medical data, healthcare expenses and healthcare insurance fund data, and personal health management data. Applications developed by third-party developers based on market demand can be directly released to users on the cloud service platform, or services to support the background of the cloud service platform management system.

In the third stage, the pharmaceutical provider is allowed to sell prescription drugs to patients online. The manufacturers of the healthcare equipment could sell healthcare equipment online to patients and all-level hospitals. MSCP provides the openness of technological interfaces and freely accessible data on cloud service platforms to all actors in the healthcare service ecosystem. Third-party developers develop healthcare applications which meet market demand, in turn, improve the quality of services provided on the platform. The government healthcare administrations will provide policy support, strengthening healthcare quality management, healthcare procedure assessment, and healthcare outcome assessment going forward using MSCP in building a self-sustaining online ecosystem. The government health administrations at all levels of cities, districts, and county administration can provide health management services through dynamic collection, analysis, and evaluation of residents' daily health data, control the spread of epidemics more quickly by monitoring infectious diseases early on, at the same time, scientifically assess healthcare expenses, quality measures, control healthcare facility costs, and ensure healthcare service quality. The healthcare insurance agencies may formulate the reimbursement policy for drugs on the market based on the outcome of treatment and its corresponding social and economic benefits, detect fraud risks, waste, and abuse of resources, and help speed up the healthcare insurance claim process. The patients could get more convenient and efficient service from other actors in the healthcare service ecosystem.

MSCP will eventually form a self-evolving and self-enriching healthcare service ecosystem of its own, leading to new wisdom in the realm of healthcare applications and business models. The establishment of the healthcare service ecosystem can free up precious healthcare resources and push efficiency to a greater extent, helping to solve problems within healthcare equality applications and business models. The establishment of the healthcare service ecosystem can free up precious healthcare resources and push efficiency to a greater extent, helping to solve problems within healthcare equality.

After the patient completes the online diagnosis and treatment process, purchases the medicine directly online according to the electronic prescription, it is very convenient. (Pharmaceutical provider)

SMCP have opened to healthcare equipment suppliers, including sensor technology manufacturers and health measurement equipment manufacturers to provide more accurate and higher-quality services for hospitals at all levels. (Manufacturers of the healthcare equipment)

Relying on the open interface of the healthcare service platform, openness of network data, and so on, healthcare applications with nuanced functions may be developed in line with demand. These applications can be released directly to members of the service platform or simply utilize the service platform as back-end infrastructure. (Third-party developers)

The current direction of development of healthcare services should be from high-quality healthcare resources concentrated in the large-scale and high-level hospitals to the medium-scale basic hospitals, ending at the small-scale network healthcare clinics. (Government Officers)

The healthcare service industry has gradually changed its direction from government control to the market. (Government Officers)

Collaboration between doctors and hospitals is seen as a means of improving the quality and efficiency of care, which can create a personalized experience for patients. (Government Officers)

The process of providing healthcare services starts with traditional treatment as the core, and then, it gradually develops to include the entire industrial healthcare chain such as prevention and rehabilitation. (Government Officers)

We can obtain medical data from the MSCP and develop the new insurance products and find new customers. (Manager of the healthcare insurance agencies)

The medical data can help us promptly detect fraud risks, waste, and abuse of resources and speed up the healthcare insurance claim process. (Manager of the healthcare insurance agencies)

The cost of health management services will be paid by all levels of government, along with a gradual increase in the number of the small-scale network healthcare clinic associated with telemedicine technology, which is expected to account for 65% of the total healthcare costs. (Government Officers)

## 4. Discussion: The Collaboration between the Relevant Actors to Enable a Healthcare Service Ecosystem

The study discusses below how the relevant actors of the ecosystem address complex issues in healthcare in a coordinated manner. It offers important implications for designing healthcare MSCPs which may help transform the healthcare industry by suggesting a blueprint for the implementation of a sustainable healthcare service ecosystem.

**Figure 2:**
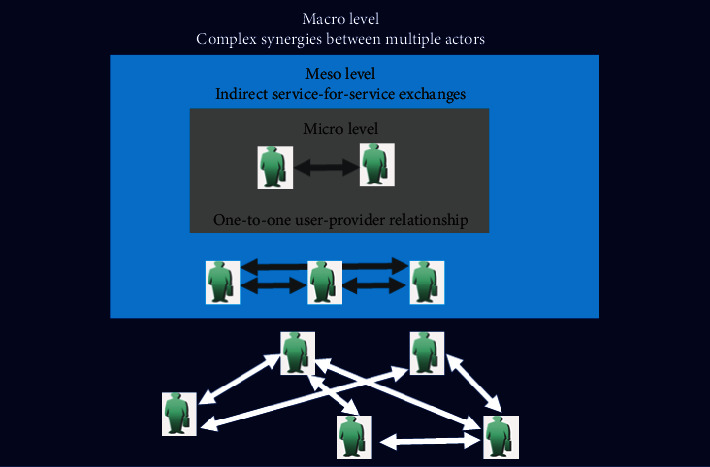
The service ecosystem levels.

The service ecosystem is proposed to consider as the micro-, meso-, and macrolevels which are shown in Figure 2, in which the interacting actors share and exchange their resources to adapt to the environment and coevolve [8]. The structure of the service ecosystem was constantly changed and adapted to achieve a long-lasting well-being. At the microlevel, interactions mainly involve the one-to-one user-provider relationship, which may be understood as a direct service-for-service exchange. At the mesolevel, indirect service-for-service exchanges occur, which are realized through triadic relationships between dyads of actors [9–11]. Synergies between multiple actors may occur in the macrolevel, and all actors comply with specific norms and rules to establish a cooperative environment [12].

In the studied case, this paper has documented the emergence and evolution of the MSCP-enabled healthcare service ecosystem, which evolved in three phases and integrated relevant actors in complementary fields, including patients, healthcare service providers, health insurance agencies, and government health administrations [13]. Among them, the healthcare service providers include online doctors, local doctors, pharmaceutical provider, and manufacturers of the healthcare equipment and third-party developers of healthcare cloud services. All actors in the MSCP-enabled healthcare service ecosystem in China have impacting factors at three levels.

At the microlevel, the actors include patients, local doctors, and online doctors. According to Akaka and Chandler [14], interactions mainly involve the one-to-one user-provider relationship. Patients (aka the “user”) are the heart of the healthcare service ecosystem, who utilize the applications provided by the platform to establish effective relationships such as the communication and exchange mechanism with the doctor online. Healthcare service providers (“Provider,” in this stage) include online doctors and local doctors. Understanding the roles and responsibilities of resource-sharing actors is important in appreciating current and potential relationships within the ecosystem. From an ecosystem perspective, there is a certain degree of interdependence between service providers and users; in this case, the local doctors and the online doctors are responsible for providing patients with various healthcare services by MSCP's application, simplify the diagnosis and treatment process, relieve the doctor's work pressure, and address a critical need of the patients; a cooperative relationship can be established to cope with illness, thus participating in the design and delivery of care for future iterations [15]. At the same time, the patients' satisfaction is regarded as a measure of whether MSPC is a net-positive system. The MSCP-enabled healthcare ecosystem is sustainable when it is designed at the microlevel.

At the mesolevel, drawing on the conceptual framework proposed by Tsiotsou [11], indirect service-for-service exchange is concerned. The actors include pharmaceutical provider, manufacturers of the healthcare equipment, and third-party developers of healthcare cloud services, who are responsible for providing patients with various healthcare service applications directly and have close interactions with patients. With the reduction of intermediate links, more benefits are obtained, the demand of the actors at mesolevel is met through the MSCP, the viability of the business model received further pragmatic validation, and MSCP is proved to be more sustainable.

At the macrolevel, the multiple participants may be expanded to involve government health administrations, and health insurance agencies [16]. They determine funding allocation and healthcare policy formulation, which can impact the operations of healthcare organizations. The main purpose of these actors is to increase the friendliness of the healthcare service ecosystem and make the effective and rational use of healthcare resources by patients. These actors directly influence the behaviors of patients and healthcare service providers at both the micro- and mesolevels. If the effects are positive, it will stimulate these agencies to adhere to an ecosystem perspective at the strategic and operational level, smoothing the transition going forward [17]. As the scale of the healthcare service ecosystem continues to grow, each actor in the ecosystem has gained more value, further enhancing its sustainability.

Practice actors at three levels interact to build the MSCP-enabled healthcare service ecosystem. In each level, the number and types of actors engaged in healthcare services, as well as the breadth of services provided, differ. Multiple elements of the ecosystem have progressively evolved and reinforced one another to create a dynamic system. The case MSCP ecosystem in China is economically sustainable and scalable and can accelerate transformation.

## 5. Conclusion

The case study suggests that the mobile smart healthcare cloud platform could enable the development of an ecosystem capable of unleashing multipronged, integrated improvements to address complex problems, which would help address the increasingly serious service crisis and promote the evolution and restructuring of the healthcare industry in developing countries. The case study offers insights into the evolution of the MSCP, enabling a healthcare service ecosystem of key actors and their impact. This paper offers an empirical inquiry into the process of healthcare ecosystem evolution and its consequences in an effort to uncover the value added by technologically enhanced healthcare services, which is based on Internet ICTs, at the same time, which has conducted the healthcare service ecosystem to direct the future medical model and shift focus towards development. In future research, we should focus on the formation of such an ecosystem, which requires efforts from multiple actors (e.g., patients, doctors, healthcare providers, insurance companies, and government healthcare administrations); actors who operate within the healthcare system create value at different ecosystem levels; they strengthen each other through their cooperation, each leveraging its core competencies, but doing so in collaboration with others for self-sustaining, maximum impact.

## Figures and Tables

**Table 1:** Interview arrangements.

Interviewees	Interview topics
10 patients	Patient satisfaction as a measure of whether a mobile health ecosystem is a net-positive system
6 healthcare service providers	Relevant technologies for each mode of healthcare service delivery and their relationship with other actors within the platform
4 developers of the SMCP	Key inflection points in the strategy, and each supporting technology and the stage of the activity
2 managers of health insurance agency	The positive elements for cooperating with the healthcare service platform
6 government officers	Their motivation and demand for setting up the healthcare service platform

## Data Availability

The datasets used and/or analyzed during the current study are available from the corresponding author on reasonable request.
